# Lightweight ProteinUnet2 network for protein secondary structure prediction: a step towards proper evaluation

**DOI:** 10.1186/s12859-022-04623-z

**Published:** 2022-03-22

**Authors:** Katarzyna Stapor, Krzysztof Kotowski, Tomasz Smolarczyk, Irena Roterman

**Affiliations:** 1 Department of Applied Informatics, Silesian University of Technology, Akademicka 16, 44-100 Gliwice, Poland; 2 Department of Bioinformatics and Telemedicine, Jagiellonian University Medical College, Medyczna 7, 30-688 Kraków, Poland

**Keywords:** Protein secondary structure prediction, U-Net, Deep learning, PSSM, HHblits

## Abstract

**Background:**

The prediction of protein secondary structures is a crucial and significant step for ab initio tertiary structure prediction which delivers the information about proteins activity and functions. As the experimental methods are expensive and sometimes impossible, many SS predictors, mainly based on different machine learning methods have been proposed for many years. Currently, most of the top methods use evolutionary-based input features produced by PSSM and HHblits software, although quite recently the embeddings—the new description of protein sequences generated by language models (LM) have appeared that could be leveraged as input features. Apart from input features calculation, the top models usually need extensive computational resources for training and prediction and are barely possible to run on a regular PC. SS prediction as the imbalanced classification problem should not be judged by the commonly used Q3/Q8 metrics. Moreover, as the benchmark datasets are not random samples, the classical statistical null hypothesis testing based on the Neyman–Pearson approach is not appropriate.

**Results:**

We present a lightweight deep network ProteinUnet2 for SS prediction which is based on U-Net convolutional architecture and evolutionary-based input features (from PSSM and HHblits) as well as SPOT-Contact features. Through an extensive evaluation study, we report the performance of ProteinUnet2 in comparison with top SS prediction methods based on evolutionary information (SAINT and SPOT-1D). We also propose a new statistical methodology for prediction performance assessment based on the significance from Fisher–Pitman permutation tests accompanied by practical significance measured by Cohen’s effect size.

**Conclusions:**

Our results suggest that ProteinUnet2 architecture has much shorter training and inference times while maintaining results similar to SAINT and SPOT-1D predictors. Taking into account the relatively long times of calculating evolutionary-based features (from PSSM in particular), it would be worth conducting the predictive ability tests on embeddings as input features in the future. We strongly believe that our proposed here statistical methodology for the evaluation of SS prediction results will be adopted and used (and even expanded) by the research community.

**Supplementary Information:**

The online version contains supplementary material available at 10.1186/s12859-022-04623-z.

## Background

The function of a protein is correlated with its tertiary structure, also known as the native structure which is a unique, stable, and kinetically accessible three-dimensional structure [1]. The first tertiary structure was determined for myoglobin by John Kendrew and his associates in 1957 [2]. For the studies on the structure of globular proteins, Kendrew received the Nobel Prize in Chemistry in 1962. More than 60 years later, there are 177 426 protein structures deposited in the Protein Data Bank [3] as of May 9th, 2021. For comparison, UniProtKB/Swiss-Prot database, which contains manually annotated and reviewed protein sequence (primary structure) has 564 638 sequences deposited and UniProtKB/TrEMBL, which contains automatically annotated and not reviewed sequences, has 214 406 399 sequences deposited as of May 9th, 2021 (The UniProt Consortium, 2021). The cost of determining sequence is significantly lower compared to the cost of determining the structures [4]. Hence, researchers try to create statistical or machine learning that would predict the structure of the proteins.

For the secondary structure prediction, three generations of methods and algorithms are described in the literature [5]. The first generation, represented by Chou-Fasman’s method, was leveraging statistical propensities of amino acids residues towards a specific secondary structure class [6]. The prediction accuracy of such methods was usually less than 60%.

The second generation of methods started in the 1980s and was leveraging sophisticated statistical methods, machine learning techniques as well as information about the neighboring residues usually using a sliding window approach [5]. It was represented by methods like GOR [7] or Lim [8], but the Q3 accuracy was still less than 65% [9].

The third generation of methods could be characterized especially by deep neural networks and additional features based on multiple sequence alignment profiles (i.e., PSSM—position-specific scoring matrices [10]) or HHblits (iterative protein sequence searching by profile hidden Markov models) [11]. The accuracy of those methods reached 80% Q3 for models such as PSIPRED [12]. Given the growing number of known protein sequences, and more effective neural network architectures, recent methods are able to predict the secondary structure with more than 70% accuracy on the 8-class problem like NetSurfP-2.0 (71.43% Q8 on CASP12) [13], SPOT-1D (73.67% Q8 on CASP12) [14] based on long short-term memory (LSTM) bi-directional recurrent neural networks (BRNN) or SAINT (74.17% Q8 on CASP12) [15] based on convolutions with the self-attention mechanism.

The next only recently emerging generation of methods, protein Language Models (LM), is inspired by advancements in the natural language processing (NLP) field [16]. The fundamental elements of these methods are sequence *embedding*s like the ones extracted from sequence-to-vector [17] or transformers [18–20] that are designed to encode some of the *grammar* of the *language of life*. One of these models, namely ProtT5-XL-U50 [19], helped to achieve the SS predictions close to NetSurfP-2.0 results (which is worse than SPOT-1D and SAINT). Importantly, the sequence embeddings can be generated in a fraction of the time with respect to evolution-based feature extraction methods like PSSMs or HHblits. The most recent success of AlphaFold2 [21] proved that NLP-inspired mechanisms like attention and transformers may be extremely useful in protein structure prediction, but the main limitation is that the training of these models needs substantial computing resources.

In this study, we present ProteinUnet2, a significantly extended and improved version of ProteinUnet, our previous deep neural network architecture for SS3 and SS8 prediction from a single sequence [22]. It is now possible to feed any number of features to the input of the network (here, we used evolutionary-based features). We performed the analysis of the significance of the input features resulting in the selection of their best combination. The architecture has been improved with the addition of attention and dropout layers and training with a variable learning rate. We designed it to be lightweight by keeping a relatively low number of parameters and using easily parallelizable convolutional layers. This new architecture allowed us to keep the prediction times lower than for predictors SAINT and SPOT-1D while maintaining similar or better performance on the benchmark datasets TEST2016, TEST2018, and CASP12. However, it should be remembered that the prediction time does not include the relatively long time of calculating the protein input features (i.e., PSSMs, HHblits, and SPOT-Contact).

For reference only, we included in Additional file 1 the comparison with the brand new AlphaFold2 (using secondary structures parsed from the predicted 3D structure) on the CASP14 dataset. For the same dataset, we also included a comparison with the ProtT5-XL-U50 language model. An in-depth comparison of our architecture with LMs (taking embeddings as input, not PSSMs or HHblits) will be the subject of a separate publication.

For the first time (to our knowledge), we raise the problem of the incorrect methodology used for prediction efficiency assessment in the previously published works. The SS prediction is a heavily imbalanced classification problem and should not be judged using commonly used Q3/Q8 metrics. Instead, we proposed to use the Adjusted Geometric Mean (AGM) metric [23], which has been proven to be more appropriate for bioinformatics imbalanced classification problems [24]. One cannot fail to mention the SOV (Segment Overlap Measure) metric (i.e., the average overlap between the observed and the predicted segments instead of the average per-residue accuracy) for the evaluation of SS prediction. The previous definitions of SOV scores (SOV'99) [25] and (SOV'94) [26] have recently been refined by improved assignment of allowance in SOV'99, which can ensure that the amount of allowance is incremental when one more element in the predicted sequence is further predicted accurately [27]. This relatively new metric requires a separate investigation of its sensitivity to imbalance classification and will be not considered here. Moreover, as the benchmark datasets are not random samples, the classical null hypothesis significance testing using the Neyman-Pearson inference approach should not be used. We propose the new assessment methodology based on the Fisher–Pitman model of inference—statistical significance from the permutation tests. We also suggest supplementing such statistical significance with the practical significance measured by Cohen’s effect size. Using the proposed statistical methodology, we compared ProteinUnet2 with state-of-the-art predictors (taking as input features PSSMs and HHblits), SAINT, and SPOT-1D.

Thus, we have made the following significant contributions: (i) introduced the new statistical methodology for SS prediction performance assessment, more appropriate in highly imbalanced SS8 prediction problem, (ii) we proposed a new lightweight U-Net-based deep architecture that enabled us to achieve very short prediction times while maintaining similar or better performance than other state-of-the-art SS predictors based on evolutionary-based input features.

## Results and discussion

Like the authors of SAINT, we focused only on SS8 prediction analysis as it contains more useful information, does not depend on the SS3 mapping method, and is much more challenging to solve.

### Comparison of predictors

We directly compare ProteinUnet2 against the most recent and accurate SS8 predictors SPOT-1D and SAINT. These state-of-the-art methods have been shown to outperform other popular predictors like MUFOLD-SS [28] or NetSurfP-2.0 [13]. For the reasons stated in the “Methods” section, in the comparison of performance, we focus mainly on the Adjusted Geometric Mean (AGM) metric for each structure (Table 1) as well as the macro-averaged AGM (Table 4) to assess the overall performance. The results for Q8 (Table 2), F1 score (Table 3), precision (Additional file 1: Table S3), and recall (Additional file 1: Table S4) are also presented.

**Table 1:**
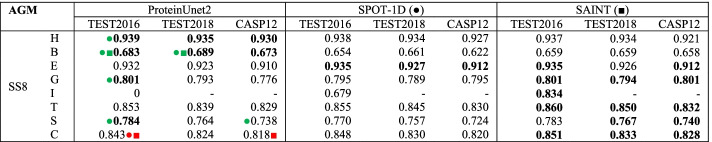
The comparison of **AGM** for each SS8 separately at the **residue level** on all test sets for ProteinUnet2 versus SPOT-1D (circle symbol) and SAINT (square symbol)

The green/red symbols on the left/right side of the ProteinUnet2 score denote the statistical significance that it has a better/worse mean at the sequence level than other networks at *p* < 0.01. The dash means the metric was impossible to calculate. The best values for each dataset and structure are boldfaced

**Table 2:**
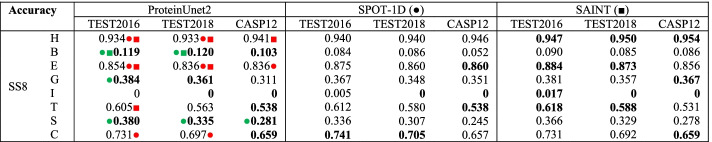
The comparison of **accuracy (Q8)** for each SS8 separately at the **residue level** on all test sets for ProteinUnet2 versus SPOT-1D (circle symbol) and SAINT (square symbol)

The symbols and boldfaced results were added similarly as in Table 1

**Table 3:**
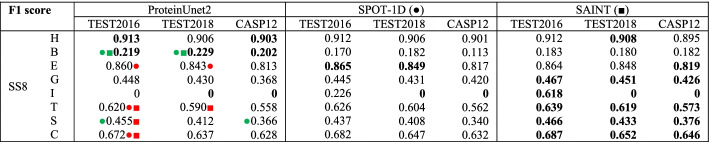
The comparison of **F1 score** for each SS8 separately at the **residue level** on all test sets for ProteinUnet2 versus SPOT-1D (circle symbol) and SAINT (square symbol)

The symbols and boldfaced results were added similarly as in Table 1

**Fig. 1:**
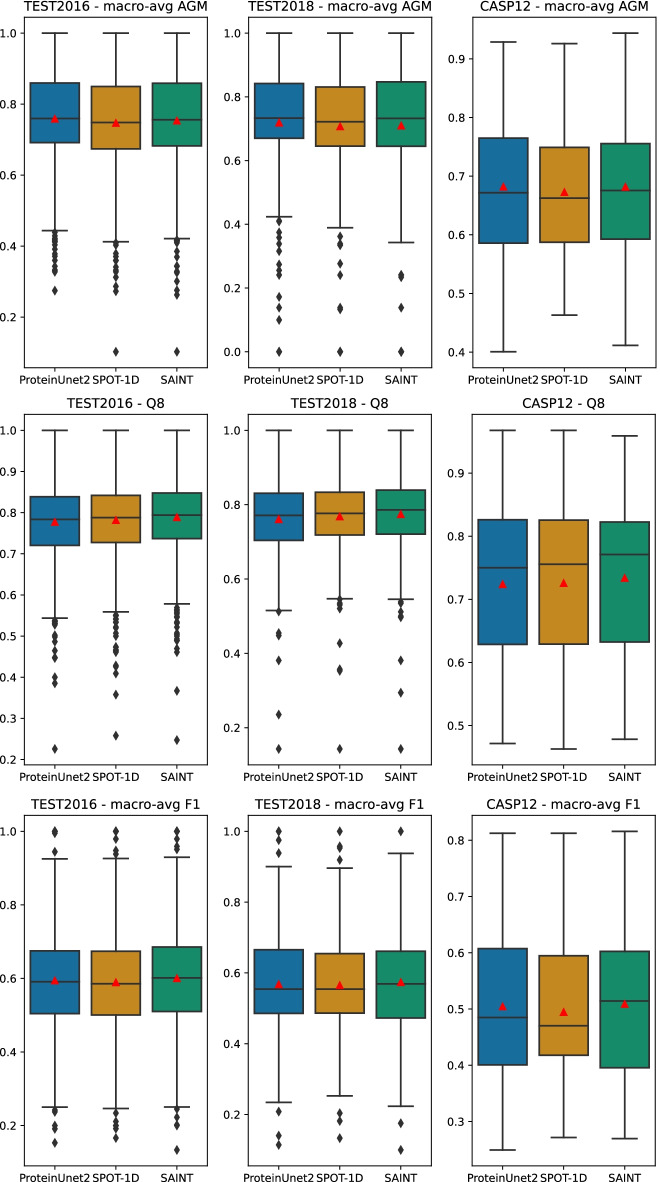
Boxplots of macro-averaged F1 (top row), macro-averaged AGM (middle row), and Q8 (bottom row) at the sequence level for TEST2016 (left column), TEST2018 (middle column), and CASP12 (right column). The red triangles denote mean scores. The exact values of means and standard deviations are given in Additional file 1: Table S5

Figure 1 presents the boxplots of macro-averaged F1 and AGM as well as Q8 metrics at the sequence level on TEST2016, TEST2018, and CASP12 datasets for 3 predictors: ProteinUnet2, SPOT-1D, and SAINT. These boxplots reveal small differences between the predictors’ medians and means (denoted by red triangles) for all presented metrics. Also, very high variability in all distributions is clearly visible. To compare quantitatively the observed slight difference, we used the statistical methodology proposed in the “Methods” section.

Tables 1 and 4 report the performances obtained for AGM metric for 8 structures and the macro-average, respectively. Table 5 presents the obtained p-values together with Cohen’s effect sizes for two separate comparisons between classifiers: ProteinUnet2 versus SPOT-1D, and ProteinUnet2 versus SAINT; on three test datasets: TEST2016, TEST2018, and CASP12.

**Table 4:**
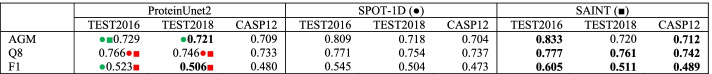
The comparison of **macro-averaged AGM, Q8,** and **macro-averaged F1** at the **residue level** on all test sets for ProteinUnet2 versus SPOT-1D (circle symbol) and SAINT (square symbol)

The symbols and boldfaced results were added similarly as in Table 1

**Table 5:**
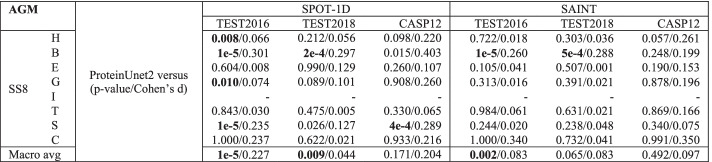
*p* values from one-sided paired permutation tests and Cohen’s d effect sizes (after the backslash) for the difference in AGM between ProteinUnet2 and other networks using the alternative hypothesis that ProteinUnet2 has a greater mean

The dash means that there were not enough samples (< 20) to run tests. *p* values lower than 0.01 are boldfaced

The obtained macro-averaged AGM results in Tables 4 and 5 prove that ProteinUnet2 has a statistically significantly higher mean than SAINT and SPOT-1D on TEST2016 dataset (*p* < 0.01). Accompanying Cohen’s effect sizes are 0.083 (very small) and 0.227 (small), respectively. ProteinUnet2 has also statistically significantly better macro-averaged AGM than SPOT-1D on TEST2018 dataset (*p* < 0.01, very small effect 0.044) while on CASP12 dataset we observe a small effect (0.204) but no significance (*p* = 0.171), probably because of the small sample size. Regarding the differences in performances on single classes (Tables 1, 5), ProteinUnet2 is significantly better (*p* < 0.01) than SAINT and SPOT-1D on rare class B on TEST2016 and TEST2018 datasets (small effect sizes 0.301, 0.297 and 0.260, 0.288, respectively). Small effect sizes are observed on this class for both classifiers on CASP12 dataset, but with no statistical significance (small sample size). ProteinUnet2 is also significantly better than SPOT-1D on classes H, G, and S on TEST2016 dataset. It is worth emphasizing that despite the lack of significance, ProteinUnet2 obtains small effect sizes (0.220 and 0.261) on class H on a small CASP12 dataset when compared with other classifiers.

In summary, when the appropriate AGM metric is used for assessment of classifiers’ performance on imbalanced SS8 prediction problem ProteinUnet2 is significantly better in overall performance (macro-averaged AGM) than SPOT-1D and SAINT on TEST2016 dataset, but with small or very small effect sizes. It is also significantly better than SPOT-1D on TEST2018 dataset and achieves comparable results with SAINT on this dataset. The comparison of ProteinUnet2 on a relatively small CASP12 dataset leads to the conclusion that there is no significant difference between our predictor and SPOT-1D nor SAINT.

For the reasons stated in the “Methods” section, we do not discuss and compare classifiers using F1 score or Q8. However, for easier comparison with the previous literature, we report the values of these metrics and statistical significance in Tables 2, 3 and 4. The effect of applying AGM is especially pronounced in terms of conclusions from statistical analysis when compared to Q8.

It is difficult not to relate the results of protein SS prediction to the more general problem of 3D protein structure prediction (from which SS can be calculated by parsing, for example using the DSSP program [29]), especially in the context of the undoubted success of AlphaFold2 on CASP14 [21]. The comparison of AlphaFold2 on the test datasets used in this study would not be fair as they were most probably used during the training phase of AlphaFold2. However, in Additional file (Additional file 1: Tables S7 and S8), we have added a separate section about the comparison of predictors on the CASP14 dataset. It includes also the results on one of the best protein language models ProtT5-XL-U50 [19] for reference. CASP14 consists of proteins selected specially for the contest and therefore they may be not a representative sample. Thus, the detailed comparative analysis of ProteinUnet2 with AlphaFold2 and language models would require training and testing on other more representative databases and input features and will be the subject of a separate publication.

### Dependence on the number of homologs

**Fig. 2:**
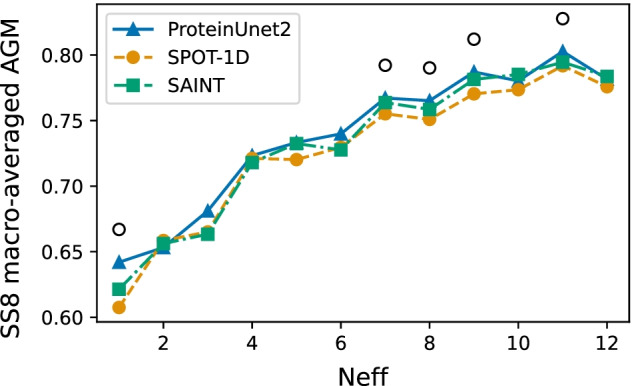
Macro-averaged AGM for predicted SS8 as a function of the number of effective homologous sequences for the TEST2016 set. The circle over the point means that ProteinUnet2 has a statistically significantly larger mean than SPOT-1D for that Neff value (one-sided paired permutation test at *p* < 0.01)

Figure 2 shows the dependence of the macro-averaged AGM on the number of effective homologous sequences (Neff) for the TEST2016 set. Each point on the plot is an average of at least 20 proteins with the given Neff (rounded down to the nearest integer) calculated by HHBlits. The figure shows that metrics increase with the increasing Neff. AGM for all networks is much lower for sequences with less than 4 homologs (Neff < 4).. The advantage of ProteinUnet2 over SPOT-1D is statistically significant (*p* < 0.01) for Neff values 1, 7, 8, 9, 11. Interestingly, this advantage is the most pronounced for Neff = 1 (AGM greater by 0.035 than SPOT-1D and by 0.021 than SAINT). ProteinUnet2 is not statistically different from SAINT in this context.

### Analysis of incorrect predictions

**Fig. 3:**
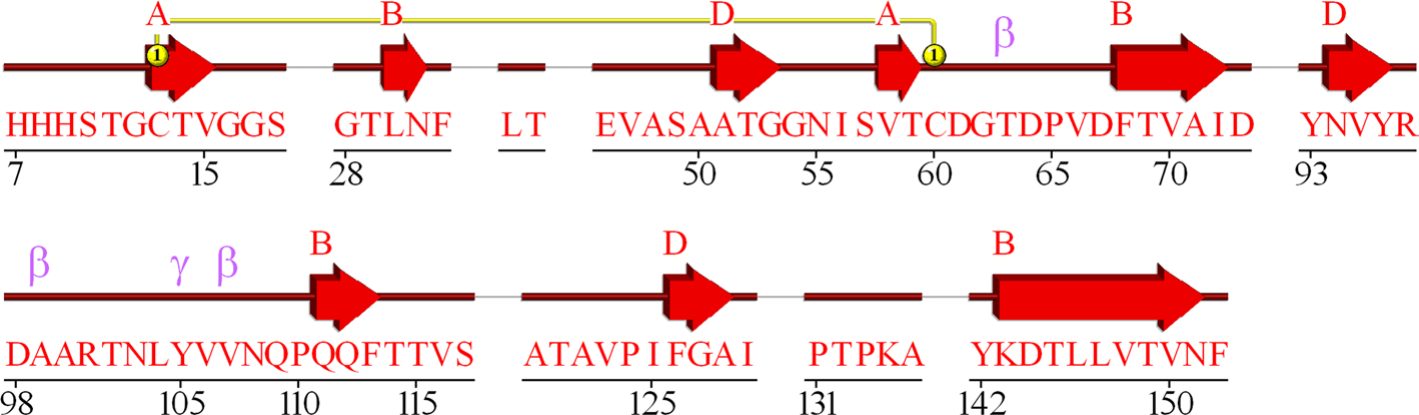
The primary and secondary structure of chain B in 5d6h protein from PDB

**Fig. 4:**
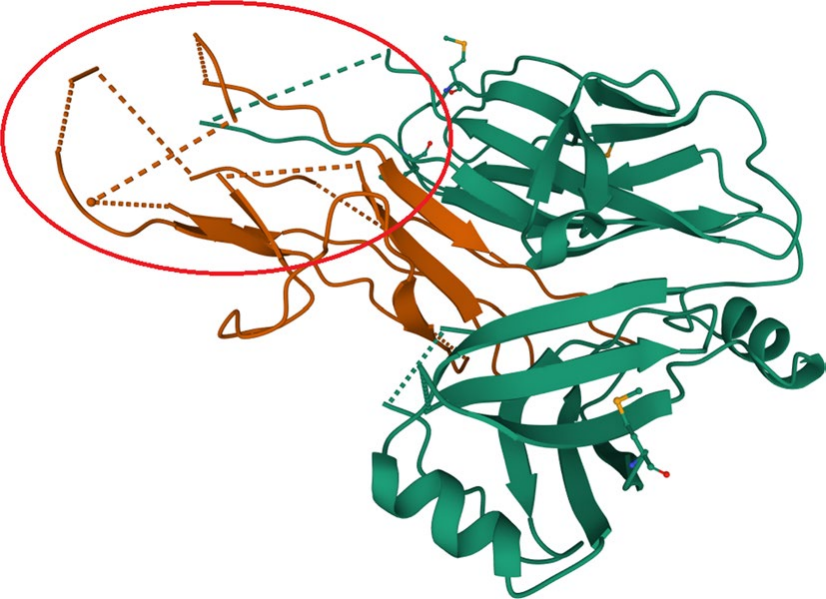
3D visualization of chain B in 5d6h protein with missing amino acids marked

We noticed that for particular sequences from TEST2016 (5doiE, 5dokA, 5d6hB) the performance of all networks is very poor (AGM < 0.3). It turned out that they are missing some amino acids in the original PDB files (5doiE—4 gaps with 35 out of 128 AA missing, 5dokA—1 gap with 34 out of 204 AA missing, 5d6hB—8 gaps with 54 out of 152 AA missing). The gaps for 5d6hB chain are presented in Fig. 3 generated using the PDBsum web server [30] and on 3D visualization from RCSB Mol Viewer [29] in Fig. 4. Even a single missing amino acid may change the secondary structure [31]. It may explain the very low performance for mentioned proteins. Thus, the problem lies in the dataset itself.

Worse results of AlphaFold2 on 4 proteins from CASP14 dataset (namely T1030, T1054, T1064, and T1099) could be explained based on FOD-M model [32]. The calculated parameters of the FOD-M model for these proteins allow us to conclude that the learning procedure used in AlphaFold2 does not take into account the so-called protein specificity [33], which is not only a function of the sequence but also of the folding environment. This problem will be studied in our future separate paper.

### Running time

Table 6 presents the inference time of ProteinUnet2, SPOT-1D, and SAINT. The times were measured on the PC with AMD Ryzen 9 3900X CPU with Nvidia RTX 3070 GPU. They do not include PSSM, HHBlits, or SPOT-Contact feature extraction times (around 28 min, 33 s, and 42 s, correspondingly, for an example protein 5ugw of length 159 according to [14]). We are focused only on improving the training and prediction times of the network itself, and we do not consider evolutionary information calculation as a part of the network. We simply compare the effectiveness of the ProteinUnet2 architecture with the state-of-the-art architectures on the same input features. The inference of ProteinUnet2 is orders of magnitude faster than SPOT-1D (up to 50 times faster for TEST2016 dataset) and around 10% faster than SAINT. A single epoch of ProteinUnet2 training takes around 2 min which gives an average of 30 min per model. The training times of SPOT-1D and SAINT were not reported but are expected to be proportionally longer.

**Table 6:** Prediction times in seconds (without time for calculating PSSMs, HHblits, and SPOT-Contact) for ProteinUnet2, SPOT-1D, and SAINT on all test sets

Dataset	ProteinUnet2	SPOT-1D	SAINT
TEST2016	229	11,796	252
TEST2018	88	3644	98
CASP12	59	486	64

## Conclusions

ProteinUnet2 significantly extends and improves our previous ProteinUnet deep architecture [22]. It introduces multiple inputs with evolutionary profiles like PSSM, HHblits, and SPOT-Contact maps. However, many other possible input features (e.g., sequence embeddings) can be easily adopted into this architecture. The performance is increased by an additional mechanism of attention and dropouts. ProteinUnet2 achieves comparable results to the state-of-the-art secondary prediction models—SPOT-1D based on LSTM-BRNN architecture and SAINT based on self-attention modules while generating predictions and training much faster than SPOT-1D and faster than SAINT. That makes it especially useful in low-end systems (low-cost GPUs or CPU-only predictions) and rapid experimentation on large datasets, assuming that the input features (like PSSM or HHblits) are already available or easy to calculate. In future work, the ProteinUnet2 architecture with learned attention layers can be further explored to interpret the mechanism of protein folding, e.g. using methods described in [33]. As bio-embeddings from [34] can be generated in a fraction of the time with respect to evolutionary-based features, we also plan to test our architecture with these input features in the near future.

The proposed methodology for assessment of the performance of secondary structure predictors based on an appropriate measure for imbalanced classification (AGM) together with permutation tests as well as analyzing significance of performance difference based on effect sizes may and should be further developed through, for example, other measures of effect size or its interpretations appropriately to the application domain.

## Methods

### Datasets

For a fair comparison, we use the same training, validation, and test datasets as SPOT-1D and SAINT. The training set TR10029 contains 10 029 proteins, and the validation set VAL983 has 983 proteins. We benchmark our model on 3 test sets: TEST2016 with 1213, TEST2018 with 250, and CASP-12 with 49 proteins. See [14, 15] for the details about these datasets. The PSSM, HHblits, and SPOT-Contact maps were provided to us by the authors of SPOT-1D (for TR10029, VAL983, TEST2016, TEST2018) and SAINT (for CASP12). The parameters used to calculate them can be found in the corresponding articles and in Additional file 1: Table S6.

### Metric for secondary structure imbalance classification problem

**Fig. 5:**
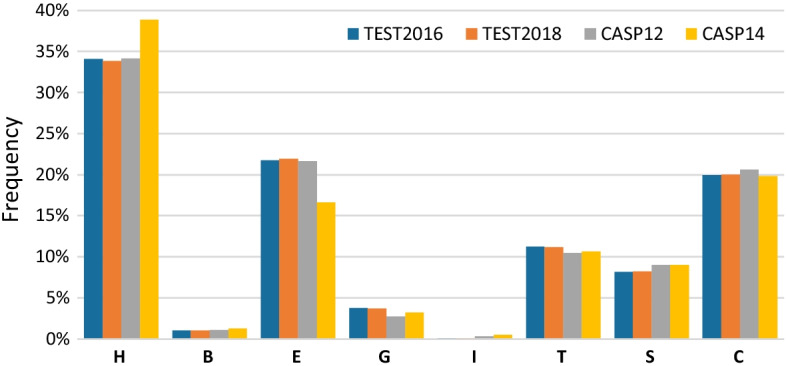
The frequencies of 8 secondary structures in TEST2016, TEST2018, CASP12, and CASP14 sets

Some protein secondary structures, e.g., alpha-helices, are much more frequent than others (Fig. 5). This leads to the class imbalance problem [35] which is rarely mentioned or addressed in the literature about SS prediction. Assessing the performance of SS classifiers plays a vital role in their construction process. The most commonly used metrics of SS prediction performance are overall accuracies Q3 and Q8 [5, 9, 36] that are not appropriate for imbalance problems [37, 38]. Using them may lead to the accuracy paradox where high accuracy is not necessarily an indicator of good classification performance [38], e.g., a classifier that always predicts class H will have ten times better accuracy than a classifier that always predicts class G (see Fig. 5).

The existing popular measures proposed for imbalanced learning like the geometric mean or F-score can still result in suboptimal models [24]. For these reasons, we used the Adjusted Geometric Mean (AGM) well-suited for bioinformatics imbalance problems [23]. It has been shown both analytically and empirically to perform better than F-score. It has no parameters (like a beta in F-score). It is given by Eq. (1) where GM is the geometric mean (Eq. 2) and N_n_ is the proportion of negative samples.

1 $$AGM=\left\{\begin{array}{ll}\frac{GM + Specificity*{N}_{n}}{1+{N}_{n}},&\quad Sensitivity>0\\ 0,&\quad Sensitivity=0\end{array}\right.$$

2 $$GM=\sqrt{Precision*Sensitivity}$$

3 $$Specificity= \frac{TN}{TN+FP}$$

4 $$Sensitivity= \frac{TP}{TP+FN}$$

5 $$Precision= \frac{TP}{TP+FP}$$

AGM’s purpose is to increase the sensitivity while keeping the reduction of specificity to a minimum. Also, the higher the degree of imbalance, the higher reaction to changes in specificity. It returns values between 0 (the worst prediction) and 1 (a perfect prediction).

We calculate AGM for each structure separately. To assess the overall quality, we use macro-averaged F1 and AGM scores. That is, we take an average of overall scores for each structure. This way we do not favor more frequent classes.

### Significance testing and effect size

Null hypothesis significance testing (nhst) is a commonly used statistical method for comparing classifier performances [38, 39] although the authors mention their caveats. In the case where the test datasets are not random (like the benchmark datasets used in the evaluation of SS prediction), using classical nhst is problematic [38]. The population model (which is the basis of nhst) is rife with assumptions that are seldom satisfied in practice and are often inappropriate for the lower levels of measurement, e.g., independence, random sampling from a parent population, an underlying Gaussian distribution for the target variable in the population, and homogeneity of variance. The permutation model is free of any distributional assumptions, does not require random sampling, is completely data-dependent, provides exact probability values, and is ideally suited for the analysis of small samples [40]. Random permutation tests based on the Fisher–Pitman model of inference [41] are thus an alternative that is strongly recommended in our case.

In our experiments, we used a one-sided paired sample permutation test for difference in mean classifier performances (perm.paired.loc function from wPerm R package). The tests are performed at the sequence level. Tests for separate structures are performed only on the subsets of sequences for which it was possible to calculate a given metric (e.g., if the structure is present in the ground truth or prediction).

Here (to our knowledge, for the first time), we propose a new methodology to compare the significance of classifier performance differences. Significance testing as well as permutation tests alone do not resolve the problem of inferential interpretation. Statistical significance shows only that an effect exists, practical significance—the effect size—shows that the effect is large enough to be meaningful in the real world. Statistical significance alone can be misleading because it’s influenced by the sample size. Increasing the sample size always makes it more likely to find a statistically significant effect, no matter how small the effect is in the real world. Effect sizes are independent of the sample size and are an essential component when evaluating the strength of a statistical claim. Some authors [42] proposed to use confidence intervals for estimation of effect size, but they require a random sample to enable inference. Cohen’s effect size d [43] that we propose to use in our study for a paired-samples can be calculated by dividing the mean difference by the standard deviation of the differences. Whether an effect size should be interpreted as negligible (d < 0.01), very small (d < 0.2), small (d < 0.5), medium (d < 0.8), or large (d < 1.2) depends on the context (application) and its operational definition [44]. Thus, we propose to report statistical significance (denoted by p-values) together with practical significance represented by effect sizes (here, Cohen’s effect size d for a paired-samples).

### ProteinUnet2 architecture

**Fig. 6:**
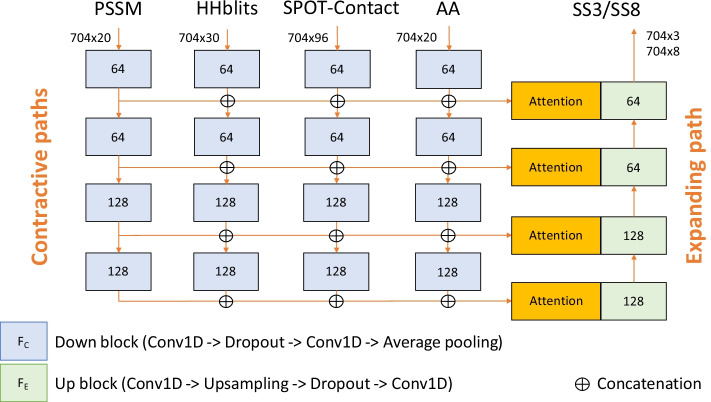
The schematic architecture of ProteinUnet2. AA is a one-hot encoded sequence of amino acids. F_C_ and F_E_ are the numbers of features in contractive and expanding paths

U-Net architectures have proven to be extremely effective in image segmentation tasks [45, 46]. The U-shaped architecture of ProteinUnet2 is based on the idea from our previous ProteinUnet for secondary structure prediction [22] (for which the results are presented in Additional file 1: Table S1). The new architecture was adjusted to handle multiple inputs by using multiple contractive paths, one for each input (Fig. 6). After each down-block, the features of all inputs are concatenated together and passed to the up-block via a skip connection. There are two output layers with softmax activations connected to the last up-block, separately for SS3 and SS8. In ProteinUnet2, we limited the maximum supported sequence length from 1024 to 704 to further improve training and inference times without losing accuracy. Anyway, SPOT-1D and SAINT were not trained with proteins longer than 700, and there are no proteins longer than 704 in our datasets. The input features and the number of filters were selected experimentally as described in the next section.

To mitigate the problem of the increased number of inputs and parameters of the network, in the final ProteinUnet2 architecture (Fig. 2), we modified the architecture to be similar to the Attention U-Net [47]. That is, we decreased the number of convolutions in each down-block from 3 to 2, added dropouts with 0.1 rate between convolutions in all blocks, and applied attention gates right before the concatenation operation. ProteinUnet2 was implemented in the environment containing Python 3.8 with TensorFlow 2.4 accelerated by CUDA 11.0 and cuDNN 8.0. The code for inference and trained models are available on the CodeOcean platform (https://codeocean.com/capsule/0425426) ensuring high reproducibility of the results. The code for training the models is accessible on demand from authors.

### Feature representation and selection

ProteinUnet2 takes a sequence of feature vectors $X=\left({x}_{1}, {x}_{2}, {x}_{3},\dots , {x}_{N}\right)$ as input, where ${x}_{i}$ is the feature vector corresponding to the *i*th residue, and it returns two sequences of structure probabilities vectors $Y=\left({y}_{1}, {y}_{2}, {y}_{3},\dots , {y}_{N}\right)$ as output, where ${y}_{i}$ is the vector of 3 or 8 probabilities of *i*th residue being in one of SS3 or SS8 states. The 8 states are specified by the secondary structure assignment program Define Secondary Structure of Proteins (DSSP) [48]. There are three helix states: 310-helix (G), alpha-helix (H), and pi-helix (I); three strand states: beta-bridge (B) and beta-strand (E); and three coil types: high curvature loop (S), beta-turn (T), and coil (C). These 8 classes are converted into the 3-class problem by grouping the states: G, H, and I into H; B and E into E; and S, T, and C into C.

Similar to SPOT-1D, our final model contains 20 features from PSSM [10], and 30 features from HHM profiles [11]. The features were standardized to ensure a 0 mean and SD of 1 in the training data. Additionally, we use contact maps generated by SPOT-Contact [49]. We use the same windowing scheme as described in SPOT-1D, but we do not standardize the contact maps as they are already in the acceptable range <0, 1>. The window size of 50 was selected experimentally based on the results from Additional file 1: Table S1 that shows F1 scores and accuracies on the largest TEST2016 set for a single ProteinUnet trained with different input features on TR10029 and validated on VAL983. Additional file 1: Table S1 suggests that SPOT-Contact features gave better results of SS8 prediction than any other input alone. The worst results are reported for 7 physicochemical properties [50]. Thus, we did not investigate them further in ProteinUnet2.

Additional file 1: Table S2 shows the F1 scores and accuracies on TEST2016 for our proposed ProteinUnet2 trained with different combinations of input features and a different number of filters in down-blocks. It reveals that SPOT-Contact features alone outperformed combined PSSM and HHblits. However, the combination of all these 3 features (keeping the same number of filters) increased F1 scores for all SS8 structures when comparing to any other feature alone. Most of our results are better for the higher number of filters, but we did not test numbers higher than 64 to avoid overfitting and to keep the number of filters in all blocks the same as in the original ProteinUnet. Thus, we decided to investigate further only the combination *PHSA 64 attention* from Additional file 1: Table S2. The architecture for this combination is presented in Fig. 6.

### Training procedures and ensembling

For the initial experiments presented in Additional file 1: Table S1 and Additional file 1: Table S2 the single models were trained on the whole TR10029 dataset and validated on VAL983. In the final ensemble, dataset TR10029 was divided into 10 stratified folds to ensure a similar ratio of each SS8 structure in each fold. There were nine factors of stratification: the sequence length—shorter/longer than mean sequence length, and one factor for each of 8 structures occurrence—fewer/more occurrences than a mean number of occurrences per chain. We trained 10 separate models, each time using different 9 folds as a training set and always using VAL983 as a validation set. The models were trained to optimize the categorical cross-entropy loss using Adam optimizer [51] with batch size 8 and an initial learning rate of 0.001. The learning rate was reduced by a factor of 0.1 when there was no improvement in the validation loss for 4 epochs. The training for each model was running until the validation loss was not improving for 7 epochs. Each time, the model with the lowest validation loss was taken. Finally, the ensemble was created from these 10 trained models by taking the average of their softmax outputs, forming the final ProteinUnet2 prediction.

## Supplementary Information

**Additional file 1.** Supplementary materials including the detailed results of ProteinUnet2 and a comparison on the CASP14 dataset.

## Data Availability

The prediction code and trained models are available on CodeOcean platform ensuring high reproducibility of the results: https://codeocean.com/capsule/0425426. The data were shared by authors of SPOT-1D and SAINT.

## Abbreviations

AGM: Adjusted geometric mean
BRNN: Bidirectional recurrent neural network
CASP: The Critical Assessment of Protein Structure Prediction
LSTM: Long short-term memory
PSSM: Position-Specific Scoring Matrix
SS3: 3-Class secondary structure
SS8: 8-Class secondary structure
