# Supplementary material for: Lightweight ProteinUnet2 network for protein secondary structure prediction: a step towards proper evaluation
Source: BMC Bioinformatics. 2022 Mar 22;23:100. doi: 10.1186/s12859-022-04623-z (PMC8939211; doi:10.1186/s12859-022-04623-z)
Supplement: Supplementary file 1 — Additional file 1. Supplementary materials including the detailed results of ProteinUnet2 and a comparison on the CASP14 dataset. [file 12859_2022_4623_MOESM1_ESM.docx]

Supplementary Material:

Lightweight ProteinUnet2 network for protein secondary structure prediction: a step towards the proper evaluation

Katarzyna Stapor^1,*,†^, Krzysztof Kotowski^1,†^, Tomasz Smolarczyk^1^ and Irena Roterman^2^

^1^Department of Applied Informatics, Silesian University of Technology, Akademicka 16, 44-100 Gliwice, Poland

^2^Department of Bioinformatics and Telemedicine, Jagiellonian University Medical College, Medyczna 7, 30-688 Krakow, Poland

*To whom correspondence should be addressed.

^†^The first two authors should be regarded as Joint First Authors

**Table S1.**The F1-score for each SS8 structure and Q8 accuracy at the residue level on TEST2016 set for a single ProteinUnet model (64 filters in the first down-block) trained on TR10029 and validated on VAL983 with different features on the input. *SPOT-Contact full* is the whole raw contact map, and the *W20/30/50* are the window sizes. The best results are bolded, the second best results are underlined.

| **F1-score** | PSSM | HHblits | SPOT-Contact full | SPOT-Contact W20 | SPOT-Contact W30 | SPOT-Contact  W50 | Physicochemical | Amino  acids |
| --- | --- | --- | --- | --- | --- | --- | --- | --- |
| H | 0.877 | 0.869 | 0.900 | 0.908 | **0.910** | **0.910** | 0.775 | 0.780 |
| B | 0.056 | 0.070 | 0 | 0.125 | 0.120 | **0.137** | 0 | 0 |
| E | 0.792 | 0.805 | **0.846** | 0.836 | 0.833 | 0.837 | 0.645 | 0.656 |
| G | 0.305 | 0.237 | 0.336 | 0.398 | 0.402 | **0.412** | 0.073 | 0 |
| I | **0** | **0** | **0** | **0** | **0** | **0** | **0** | **0** |
| T | 0.527 | 0.498 | 0.577 | **0.602** | 0.596 | 0.592 | 0.391 | 0.403 |
| S | 0.315 | 0.262 | 0.376 | **0.398** | 0.374 | **0.398** | 0.100 | 0.127 |
| C | 0.588 | 0.589 | **0.656** | 0.643 | 0.646 | 0.644 | 0.488 | 0.488 |
| Q8 | 0.702 | 0.698 | 0.746 | 0.747 | **0.748** | 0.747 | 0.592 | 0.596 |

**Table S2.**The F1-score for each SS8 structure and Q8 accuracy at the residue level on TEST2016 set for a single ProteinUnet2 model trained on TR10029 and validated on VAL983 with different combinations of features on the input: P – PSSM, H – HHblits, S – SPOT-Contact W50, A - Aminoacids. The numbers in the combination name define the number of filters in the first down-block of ProteinUnet2. The best results are bolded, the second best results are underlined. In the last row the number of trainable parameters of the network is given.

| **F1-score** | PH 64 | PS 64 | PSA 64 | PHS 16 | PHS 32 | PHS 64 | PHS 64  attention | PHSA 16 | PHSA 32 | PHSA 64 | PHSA 64  attention |
| --- | --- | --- | --- | --- | --- | --- | --- | --- | --- | --- | --- |
| H | 0.891 | 0.909 | **0.910** | 0.907 | 0.909 | **0.910** | 0.908 | **0.910** | 0.909 | 0.909 | 0.909 |
| B | 0.095 | 0.133 | 0.130 | 0.099 | 0.165 | 0.149 | 0.139 | 0.103 | 0.112 | 0.159 | **0.170** |
| E | 0.824 | 0.848 | 0.845 | 0.845 | 0.849 | 0.851 | 0.849 | 0.847 | 0.849 | 0.851 | **0.855** |
| G | 0.334 | 0.409 | 0.415 | 0.380 | 0.401 | 0.423 | 0.420 | 0.407 | 0.412 | 0.413 | **0.430** |
| I | **0** | **0** | **0** | **0** | **0** | **0** | **0** | **0** | **0** | **0** | **0** |
| T | 0.550 | 0.601 | 0.603 | 0.588 | 0.596 | 0.604 | 0.608 | 0.597 | 0.595 | 0.604 | **0.613** |
| S | 0.312 | 0.414 | 0.428 | 0.394 | 0.417 | 0.419 | 0.387 | 0.409 | 0.431 | **0.437** | 0.433 |
| C | 0.619 | 0.658 | 0.661 | 0.654 | 0.661 | 0.662 | 0.664 | 0.657 | 0.656 | 0.664 | **0.668** |
| Q8 | 0.723 | 0.755 | 0.754 | 0.751 | 0.754 | 0.756 | 0.758 | 0.753 | 0.754 | 0.757 | **0.761** |
| Parameters | 2’705k | 2’734k | 3’838k | 895k | 1’588k | 3’843k | 4’047k | 1’026k | 1’946k | 4’946k | 5’373k |

**Table S3.**The comparison of precision for each SS8 separately at the residue level on all test sets for ProteinUnet2, SPOT-1D, and SAINT. The dash means the metric was impossible to calculate.

| **Precision** | | ProteinUnet2 | | | SPOT-1D | | | SAINT | | |
| --- | --- | --- | --- | --- | --- | --- | --- | --- | --- | --- |
|  |  | TEST2016 | TEST2018 | CASP12 | TEST2016 | TEST2018 | CASP12 | TEST2016 | TEST2018 | CASP12 |
| SS8 | H | 0.892 | 0.880 | 0.865 | 0.884 | 0.874 | 0.856 | 0.879 | 0.868 | 0.839 |
|  | B | 0.536 | 0.516 | 0.545 | 0.671 | 0.640 | 0.667 | 0.760 | 0.659 | 0.769 |
|  | E | 0.864 | 0.844 | 0.787 | 0.852 | 0.834 | 0.774 | 0.843 | 0.819 | 0.780 |
|  | G | 0.520 | 0.505 | 0.415 | 0.547 | 0.537 | 0.481 | 0.581 | 0.577 | 0.469 |
|  | I | 1 | - | - | 1 | - | - | 1 | - | - |
|  | T | 0.636 | 0.618 | 0.580 | 0.641 | 0.628 | 0.588 | 0.663 | 0.653 | 0.620 |
|  | S | 0.565 | 0.531 | 0.528 | 0.624 | 0.602 | 0.559 | 0.639 | 0.629 | 0.582 |
|  | C | 0.622 | 0.587 | 0.599 | 0.631 | 0.599 | 0.609 | 0.648 | 0.617 | 0.633 |

**Table S4.**The comparison of recall for each SS8 separately at the residue level on all test sets for ProteinUnet2, SPOT-1D, and SAINT.

| **Recall** | | ProteinUnet2 | | | SPOT-1D | | | SAINT | | |
| --- | --- | --- | --- | --- | --- | --- | --- | --- | --- | --- |
|  |  | TEST2016 | TEST2018 | CASP12 | TEST2016 | TEST2018 | CASP12 | TEST2016 | TEST2018 | CASP12 |
| SS8 | H | 0.935 | 0.934 | 0.945 | 0.941 | 0.941 | 0.949 | 0.948 | 0.952 | 0.958 |
|  | B | 0.138 | 0.147 | 0.124 | 0.097 | 0.106 | 0.062 | 0.104 | 0.104 | 0.103 |
|  | E | 0.856 | 0.841 | 0.841 | 0.878 | 0.865 | 0.865 | 0.887 | 0.878 | 0.861 |
|  | G | 0.393 | 0.374 | 0.331 | 0.375 | 0.360 | 0.373 | 0.390 | 0.370 | 0.390 |
|  | I | 0 | 0 | 0 | 0.128 | 0 | 0 | 0.447 | 0 | 0 |
|  | T | 0.606 | 0.565 | 0.538 | 0.612 | 0.581 | 0.538 | 0.618 | 0.589 | 0.532 |
|  | S | 0.381 | 0.336 | 0.281 | 0.337 | 0.309 | 0.245 | 0.367 | 0.330 | 0.278 |
|  | C | 0.731 | 0.697 | 0.659 | 0.741 | 0.705 | 0.657 | 0.731 | 0.692 | 0.659 |

**Table S5.**The comparison of macro-averaged F1, macro-averaged AGM, and Q8 at the residue level on all test sets for ProteinUnet2 vs SPOT-1D (circle symbol) and SAINT (square symbol).

|  | ProteinUnet2 | | | SPOT-1D | | | SAINT | | |
| --- | --- | --- | --- | --- | --- | --- | --- | --- | --- |
|  | TEST2016 | TEST2018 | CASP12 | TEST2016 | TEST2018 | CASP12 | TEST2016 | TEST2018 | CASP12 |
| F1 | 0.594 ±0.129 | 0.568 ±0.151 | 0.499 ±0.137 | 0.590 ±0.130 | 0.565 ±0.148 | 0.495 ±0.132 | 0.601 ±0.132 | 0.574 ±0.148 | 0.509 ±0.136 |
| AGM | 0.759 ±0.123 | 0.719 ±0.175 | 0.682 ±0.133 | 0.747 ±0.127 | 0.707 ±0.176 | 0.673 ±0.123 | 0.754 ±0.127 | 0.710 ±0.182 | 0.682 ±0.133 |
| Q8 | 0.777 ±0.092 | 0.761 ±0.115 | 0.724 ±0.125 | 0.782 ±0.092 | 0.768 ±0.111 | 0.726 ±0.127 | 0.789 ±0.090 | 0.774 ±0.110 | 0.734 ±0.122 |

**Table S6.**Parameters for calculating PSSM and HHblits features as given by the authors od SPOT-1D and MULTICOM2.

| **Parameter** | **SPOT-1D** | **SAINT** | **MULTICOM2** |
| --- | --- | --- | --- |
| PSI-Blast iterations | 3 | 3 | 3 |
| Reference database for PSSM | UniRef90 | UniRef90 | UniRef90 |
| HHsuite version | 3.0.3 | 3.X.X | 3.2.0 |
| Reference database for HHblits | Uniprot20 (10/2017) | Uniprot20 (03/2013) | UniProt20 (02/2016) |
| Link to the generated profiles | https://servers.sparks-lab.org/downloads/SPOT-1D-dataset.tar.gz | https://drive.google.com/drive/folders/1reklYZ39SL_iSRJzHYkvMD8zuidSnRc7 | https://github.com/multicom-toolbox/multicom/tree/multicom_v2.0 |

# Results on CASP14

**Table S7.**The comparison of macro-averaged AGM on 30 proteins from CASP14 for ProteinUnet2, AlphaFold2, and ProtT5-XL-U50. The best results per protein are bold-faced, the second best are underlined.

| **Protein name** | **ProteinUnet2** | **SAINT** | **SPOT-1D** | **AlphaFold2** | **ProtT5-XL-U50** |
| --- | --- | --- | --- | --- | --- |
| T1024 | 0.507 | 0.712 | **0.726** | 0.520 | 0.549 |
| T1026 | 0.629 | 0.722 | 0.710 | **0.733** | 0.694 |
| T1027 | 0.560 | 0.582 | 0.587 | 0.589 | **0.609** |
| T1029 | 0.779 | 0.884 | **0.890** | 0.798 | 0.536 |
| T1030 | 0.806 | 0.607 | 0.595 | 0.607 | **0.886** |
| T1031 | 0.630 | **0.779** | 0.745 | 0.707 | 0.668 |
| T1032 | 0.636 | **0.838** | 0.803 | 0.733 | 0.706 |
| T1033 | 0.266 | 0.490 | 0.496 | **0.692** | 0.299 |
| T1035 | 0.397 | 0.223 | 0.251 | **0.798** | 0.430 |
| T1037 | 0.452 | 0.673 | 0.683 | **0.715** | 0.681 |
| T1038 | 0.514 | 0.532 | 0.544 | **0.655** | 0.534 |
| T1039 | 0.461 | 0.611 | 0.625 | 0.626 | **0.627** |
| T1040 | 0.350 | 0.183 | 0.204 | **0.448** | 0.435 |
| T1041 | 0.461 | 0.589 | 0.582 | **0.698** | 0.496 |
| T1042 | 0.699 | 0.456 | 0.564 | **0.804** | 0.580 |
| T1043 | 0.482 | 0.469 | 0.467 | **0.710** | 0.515 |
| T1046s1 | 0.514 | 0.709 | 0.709 | **0.786** | 0.433 |
| T1046s2 | 0.822 | 0.890 | 0.901 | **0.927** | 0.724 |
| T1049 | 0.584 | 0.627 | 0.630 | **0.755** | 0.536 |
| T1054 | 0.769 | 0.717 | 0.741 | 0.715 | **0.855** |
| T1056 | 0.663 | 0.698 | 0.695 | **0.828** | 0.601 |
| T1064 | 0.648 | 0.662 | **0.668** | 0.634 | 0.499 |
| T1067 | 0.664 | 0.670 | 0.691 | **0.737** | 0.695 |
| T1073 | 0.581 | 0.885 | 0.885 | 0.889 | **0.924** |
| T1074 | 0.529 | **0.796** | 0.669 | 0.607 | 0.557 |
| T1079 | 0.694 | 0.639 | 0.730 | **0.927** | 0.857 |
| T1080 | 0.693 | 0.398 | 0.396 | 0.767 | **0.810** |
| T1082 | 0.366 | 0.631 | 0.645 | **0.707** | 0.428 |
| T1090 | 0.643 | 0.682 | 0.666 | **0.736** | 0.657 |
| T1099 | 0.718 | 0.770 | **0.785** | 0.657 | 0.464 |

We compared ProteinUnet2 in terms of SS8 prediction with AlphaFold2 on the set of 34 proteins for which the PDB targets were available on the official CASP14 challenge page ([predictioncenter.org/download_area/CASP14/targets](https://predictioncenter.org/download_area/CASP14/targets/casp14.targets.T-dom.public_11.29.2020.tar.gz)) excluding 4 proteins longer than 704 residues. For the same targets, we calculated predictions for ProtT5-XL-U50 [1] using the online server by [2] (<https://embed.protein.properties/>). The PSSM and HHblits profiles for ProteinUnet2, SAINT, and SPOT-1D predictions were obtained from the authors of MULTICOM2 [3] (exact parameters are given in Supplementary Table S6). We calculated SPOT-Contact maps (for ProteinUnet2, SAINT, and SPOT-1D) using the code shared by the authors (<https://servers.sparks-lab.org/downloads/SPOT-Contact_local.tgz>).

The macro-AGM per protein is presented in Supplementary Table S7. The results for SS8 prediction in terms of Q8/macro-F1/macro-AGM at the residue level are presented in Supplementary Table S8. AlphaFold2 achieved the best results for 17 out of 30, and ProtT5-XL-U50 for 6 out of 30 proteins. The AGM results for ProteinUnet2, SAINT, and SPOT-1D are much lower than for AlpfaFold2 and ProtT5-XL-U50. In Q8 and F1, only AlphaFold2 stands out strongly from the others. However, it should be noted that both AlphaFold2 and ProtT5-XL-U50 were trained on the orders of magnitude larger datasets and substantial computational resources what makes the comparison unfair. Still, ProteinUnet2 achieved higher macro-averaged AGM than AlphaFold2 for 4 proteins in CASP14 (T1030, T1054, T1064, and T1099), and for 8 proteins in comparison to ProtT5-XL-U50 (T1029, T1042, T1046s1, T1046s2, T1049, T1056, T1064, T1099). ProteinUnet2 was better for 8 proteins than SAINT (T1030, T1035, T1040, T1042, T1043, T1054, T1079, T1080) and for 7 proteins than SPOT-1D (T1030, T1035, T1040, T1042, T1043, T1054, T1080).

**Table S8.** The comparison of **macro-averaged AGM, Q8,** and **macro-averaged F1** at the **residue level** on CASP14 for ProteinUnet2 SAINT, SPOT-1D, AlphaFold2, and ProtT5-XL-U50. The best results are bold-faced, the second best are underlined.

| CASP14 | ProteinUnet2 | SAINT | SPOT-1D | AlphaFold2 | ProtT5-XL-U50 |
| --- | --- | --- | --- | --- | --- |
| AGM | 0.643 | 0.679 | 0.680 | **0.830** | 0.763 |
| Q8 | 0.618 | 0.689 | 0.683 | **0.726** | 0.686 |
| F1 | 0.357 | 0.425 | 0.426 | **0.557** | 0.446 |

# References

[1] A. Elnaggar *et al.*, “ProtTrans: Towards Cracking the Language of Lifes Code Through Self-Supervised Deep Learning and High Performance Computing,” *IEEE Trans. Pattern Anal. Mach. Intell.*, pp. 1–1, 2021, doi: 10.1109/TPAMI.2021.3095381.

[2] C. Dallago *et al.*, “Learned Embeddings from Deep Learning to Visualize and Predict Protein Sets,” *Current Protocols*, vol. 1, no. 5, p. e113, 2021, doi: 10.1002/cpz1.113.

[3] T. Wu, J. Liu, Z. Guo, J. Hou, and J. Cheng, “MULTICOM2 open-source protein structure prediction system powered by deep learning and distance prediction,” *Sci Rep*, vol. 11, no. 1, p. 13155, Jun. 2021, doi: 10.1038/s41598-021-92395-6.
